# Medico-Chirurgical Society

**Published:** 1879-02-20

**Authors:** 


### EDITORIAL AND MISCELLANEOUS.

### *MEDICO-CHIRURGICAL SOCIETY.*

The above Society is in a flourishing condition, but no reports have been published in our Journal for some time, by reason of unavoidable absence of the Reporter from the meetings. The Society will hereafter assemble in the beautiful and commodious hall of the Academy of Science, on Mitchell street.

The officers of the Society, elected for the present year are the following: T. S. Powell, M.D., President; J. J. Knott, M.D., First Vice-President; J. C. Olmstead, M.D., Second Vice-President; R. C. Word, M.D., Reporter; A. R. Alley, M.D., Secretary. Time of meeting, first and third Monday nights of each month.

